# Relationship between Lower Limbs Performance and Spinal Alignment in Parkinson’s Disease Patients: An Observational Study with Cross Sectional Design

**DOI:** 10.3390/jcm11133775

**Published:** 2022-06-29

**Authors:** Luciano Bissolotti, Matteo Rota, Stefano Calza, Eleuterio A. Sanchez Romero, Andrea Battaglino, Jorge H. Villafañe

**Affiliations:** 1 Rehabilitation Service, Fondazione Teresa Camplani Casa di Cura Domus Salutis, 25123 Brescia, Italy; luciano.bissolotti@ancelle.it; 2 LARIN: Neuromuscular and Adapted Physical Activity Laboratory, 25123 Brescia, Italy; 3 Department of Molecular and Translational Medicine, University of Brescia, 25121 Brescia, Italy; matteo.rota@unibs.it (M.R.); stefano.calza@unibs.it (S.C.); 4 Department of Physiotherapy, Faculty of Sport Sciences, Universidad Europea de Madrid, 28670 Villaviciosa de Odón, Spain; 5 Musculoskeletal Pain and Motor Control Research Group, Faculty of Sport Sciences, Universidad Europea de Madrid, 28670 Villaviciosa de Odón, Spain; 6 Department of Physiotherapy, Faculty of Health Sciences, Universidad Europea de Canarias, 38300 Santa Cruz de Tenerife, Spain; 7 Musculoskeletal Pain and Motor Control Research Group, Faculty of Health Sciences, Universidad Europea de Canarias, 38300 Santa Cruz de Tenerife, Spain; 8 IRCCS Fondazione Don Carlo Gnocchi, 20148 Milan, Italy; abattaglino.res@gmail.com

**Keywords:** Parkinson’s disease, lower limb, spine

## Abstract

Parkinson’s disease (PD) is a progressive neurodegenerative disease determining spinal deformities and muscle rigidity, weakness and dystonia that can be related to a change in muscular output during sit-to-stand tasks (STS). Purpose: The aim of this study was to determine the impacts of spinal alignment on lower limbs performance during STS tasks in Parkinson’s disease (PD) patients and healthy controls. Methods: In total, 43 consecutive PD patients (“PD” Group, 25 males and 18 females; age 73.7 ± 7.1) and 42 people not affected by any type of neurological disease (“CON” Group, 22 males, 20 females; age 69.8 ± 6.0) participated in the observational study. The clinical assessment included: IPAQ (International Physical Activity Questionnaire), Hoehn Yahr score, plumb-line distance from the spinous process of C7, kyphosis apex and the spinous process of L3 and S1. We used the Muscle Quality Index test (MQI) to assess muscle power output during STS in both groups. Results: The MQI test measurements of absolute and relative lower limb power was significantly lower in the PD group, in addition to a negative correlation with age and a positive correlation with PL-L3 in that group of patients. Conclusions: A final consideration regarding our results leads to the possibility that the preservation of lumbar lordosis may be one of the factors for maintaining efficient biomechanics of the lower limb muscles, with the preservation of the physiological contractile characteristics of these muscles being the objective for a multidisciplinary rehabilitation based on postural exercises of the spine and a program of training exercises for the lower limb muscles.

## 1. Introduction

Patients with Parkinson’s disease (PD) often present with abnormal posture. Due to the rigidity and dystonia determined by the disease, many PD patients have deformity of their limbs, neck or trunk [1,2]. The usual deformity is the typical “stooped simian appearance”, with flexion of the hips and knees and rounding of the shoulders, but a significant subgroup of patients show more severe postural deformities in the sagittal or frontal plane, comprising camptocormia, antecollis, Pisa syndrome and scoliosis [3,4]. These severe postural deformities are often associated with significant disability and have a multifactorial pathophysiology.

Contributing factors include muscular rigidity, axial dystonia, weakness caused by myopathy, body scheme defects due to centrally impaired proprioception and structural changes in the spine [5]. These factors’ relative contribution varies among patients, and their management therefore remains complex; simultaneously, it is also widely accepted that functional impairment accompanies that of the joints and spine. [4].

It has also been reported that the strength and power of lower-limb muscles are impaired in PD patients, and that the amount of power produced by lower extremities’ muscles is predictive and related to independence in the activities of daily living [6].

The assessment of performance during repeated sit-to-stand (STS) tasks can be useful in clinical practice because of its correlation to the risk of falls and the possibility of measuring the strength and power of lower extremities in a cost-effective way [7].

In PD patients, posture and function have been investigated in terms of locomotion, sit-to-stand performance and fear of falls or occurred falls, but, to the best of our knowledge, until now, nobody has investigated the relationship between spinal alignment and lower limbs performance during repeated STS. For this purpose, we selected the Muscle Quality Index (MQI) test [8]. This test is particularly suited to evaluate lower limbs function (power production) in PD patients because it investigates a task particularly significant for daily life, which is at the same time particularly compromised in those patients [8]. MQI is indeed a time-based task, in which the examiner asks the patient to raise 10 times from a chair without manual help, and the employed time in seconds is the test outcome.

In PD patients, spine deformities can be assessed by means of clinical or radiographic parameters, and some of the measures taken during the clinical examination present a significant correlation to the sagittal balance of the spine as assessed on an X-ray examination [4,9,10]. The purpose of this study was to assess the existence of correlations among spinal alignment parameters and the power produced by lower extremity muscles during repeated STS tasks as described by the MQI test.

## 2. Materials and Methods

### 2.1. Design

This was an observational study with a cross-sectional case-control design. Each participant signed an informed consent form. The Ethical Committee of ATS Brescia approved the study (N°: NP3542). The study was performed in accordance with the ethical standards laid down in the 1964 Declaration of Helsinki and its later amendments.

### 2.2. Participants

Inclusion criteria for both PD and CON (control, healthy subjects) groups were age 64–80 years old, forbearance to a sitting position for at least 20 min and an absence of traumatic articular rigidity preventing sit-to-stand passage. In addition, to be included in the PD group, both an established PD diagnosis and an absence of pharmacological therapy changes in the previous month were necessary. Exclusion criteria for both groups comprised a presence of any other neurological diagnosis including atypical parkinsonism, a presence of persistent orthostatic hypotension intended as a drop in blood pressure greater than 30 mmHg in sit-to-stand passage, a Mini Mental Examination score lower than 23/30, a Body Mass Index larger than 34, severe visual impairment or other neurosensory disability preventing the Muscle Quality Index test execution and a recent hospital admission for profound venous thrombosis, heart stroke, heart surgery or acute heart failure within the previous three months.

In total, 43 consecutive PD patients (Group PD, 25 males and 18 females; age 73.7 ± 7.1) who were diagnosed by a neurologist according to the MDS clinical criteria for idiopathic Parkinson’s disease were included in the study protocol [11]. They were enrolled consecutively during a medical examination in a rehabilitation department and were aged 64 to 80 years (mean 71.5 ± 7.9 years). None of the participants exhibited dyskinesias.

In total, 42 healthy subjects not affected by any type of neurological disease (CON group, 22 males, 20 females; mean 71.5 ± 7.9 years) who underwent a clinical examination at Fondazione Teresa Camplani Casa di Cura Domus Salutis between January 2018 and September 2019 for non-neurological reasons were enrolled consecutively by phone calls.

All the subjects included in the PD and CON groups were maintaining their ability to walk without the help of a caregiver. The PD group followed their normal medication regimen during testing and the functional evaluation and examination was performed during the ON phase. Each participant signed an informed consent form.

### 2.3. Clinical Measurements

After signature of informed consent was given, physical activity levels were assessed for all the subjects using the International Physical Activity Questionnaire (IPAQ), and the amount of energy expended weekly in physical activities was calculated in Metabolic Equivalents (METs) [12].

As part of a multidimensional assessment model, the Hoehn and Yahr scale (range 0–5) was used to define the disease severity in patients with PD [13,14]. Subjects then underwent a clinical examination that included an assessment of body weight and standing height, as well as a calculation of body mass index [15]. This procedure was followed by a sagittal balance evaluation by plumb-line distances determination at the levels of C7 (PL-C7), kyphosis apex (PL-AK), L3 (PL-L3) and S1 (PL-S1). The trunk rotation angle (TRA, expressed in degrees) was also measured with a Bunnell scoliometer™ (Orthopedic Systems, Inc., Hayward, CA, USA) in a standing, bent-over position (arms hanging and palms together) [16]. The sagittal profile of the spine was measured by assessing the tilt with a gravity-dependent inclinometer (Isomed Inc., 975 SE Sandy Blvd, Portland, OR 97214, USA), as previously described in the literature [10].

Thereafter, the subjects performed the Muscle Quality Index test, consisting of raising 10 times from a chair without manual help. The time needed to complete the task was the outcome, and we combined it with other parameters in the following equation [8]:

MQI = [(lower limb length × 0.4) × body mass × gravity × 10]/time sit-sand.

We obtained the MQI test results in terms of employed time, absolute power and relative power (Watt and Watt/Kg body weight), which was later used in statistical analysis.

### 2.4. Statistics

A descriptive analysis was performed using absolute and relative frequencies for categorical variables and medians and interquartile range for continuous data. Between-group comparison was performed using the Chi-square test and Fisher’s exact test for categorical variables and the nonparametric Wilcoxon–Mann–Whitney test for continuous variables. The level of statistical significance was set at 0.05. The analyses were performed with SAS version 9.4 (SAS Institute Inc., Cary, NC, USA).

## 3. Results

PD patients belonged to Hoehn and Yahr class 1–2 in 53% of the cases and 2.5–3 in 47%. The mean disease duration was 5.6 ±4.3 years. PD patients were older than controls (median age 75.3 vs 68.9, *p* < 0.01) and had similar BMIs (median BMI 27.2 vs. 26.9, *p* = 0.80). The ATR showed no difference between the two groups (*p* = 0.74). According to the IPAQ questionnaire, the amount of weekly energy expenditure in exercise or non-exercise physical activity was higher in controls than in PD patients (median weekly METs 1140 vs. 910, *p* = 0.07), but this difference did not reach statistical significance. The absolute (Watt) and relative (Watt/Kg) lower-limb power measured by the MQI test was lower in the PD group (Table 1).

The mean distances of PL-C7 and PL-AK were significantly higher in PD group (*p* < 0.05), while the mean angle of upper kyphosis as measured at C7-T1 by the digital inclinometer was lower (*p* < 0.05). The S1 plumb line (PL-S1) was higher in controls (Table 2). 

A positive correlation with PL-L3 was observed in the PD group, Table 3.

In both groups, descriptive statistics were used to stratify the cohort of subjects in three different levels of performance according to the value of power measured during MQI. The quartile analysis divided the data into three points—a lower quartile (below 0.9 Watt/kg), median (from 1.0 to 1.6 Watt/kg) and upper quartile (over 1.7 Watt/kg)—to form groups within the dataset. The lower quartile, or first quartile, is denoted as the group of patients with the lowest ability to produce power with the lower limbs during sit-to-stand exercises as described by the MQI test.

Then, with a clinical purpose, the PD patients and the CON group were classified into three different classes and the threshold level of 1.7 Watt/kg was overcome by 42% in the PD patients and by 70% in the CON group, with none of this last group being below the critical level of 0.9 Watt/kg compared to 14% of the first one. Overall, 44% of the PD patients were in the 1.0–1.6 Watt/kg range compared to 30% of the CON group.

The MQI values were positively (0.35) significantly correlated with the plumb line on the spinous process of L3 in the PD group (*p* < 0.05), Table 3.

## 4. Discussion

To our knowledge, this is the first study that evaluated the performance of lower limbs in PD patients through MQI and their correlation with trunk deformity. The results of this work suggest that hip- and knee-extensor strength and power are significantly reduced in people with PD. MQI has been used in elderly people with the aim of assessing sarcopenia and/or lower extremities’ power deficiency, but in this study, we demonstrated that it can be used even in PD patients to evaluate lower-limb power during a standard clinical examination. We confirm that, as described by other authors, PD patients demonstrate a reduction in lower-limb power when compared to healthy controls [17,18,19]. 

Several studies have shown that many postural abnormalities, such as an inability to modulate the response magnitude to different postural demands or a delayed initiation or reduced scaling of voluntary postural responses, contribute to altering the dynamic performance of lower limbs in PD patients [20], but so far, neither MQI nor spinal sagittal profile of the trunk have been measured in this type of population. The results of our study suggest that the postural abnormalities of spinal alignment are another negative factor affecting sit-to-stand performance as demonstrated by the positive correlation between MQI and L3 plumb line in the PD group (*p* < 0.05). No correlations were found with the C7 plumb line or the angles measured by inclinometer at C7-T1 or T12-L1. In our opinion, this is not surprising because the existence of a correlation between the PL-L3 value and MQI has to be considered as a key step because, as described by previous papers published in the literature, in PD patients the L3 plumb-line value positively correlates with the spinopelvic angle (SPA) [10,11,21]. The spinopelvic angle has a positive correlation with lumbar lordosis and a negative one with pelvic tilt angles, as measured by a standard X-ray exam [10].

These correlations explain how the loss of lumbar lordosis in PD is accompanied by lower limb functionality impairment, and this recalls what has been described by Nakamura et al. in a recent study, where the authors demonstrated how the LL value correlates to the timed up-and-go test performance [22]. The absence of a correlation between the lower-extremity strength and the spinal alignment parameters in the control group, not affected by neurodegenerative disease, demonstrates that PD patients are affected by a specific maladaptive process that involves spinal alignment and lower limbs through the lumbopelvic unit.

As expected by the results derived by other studies [23,24,25], there is a significant negative correlation between age and MQI in PD patients: older age correlates with decreased lower-limb function. The significant correlation only present in the PD group may suggest that the negative effect of the aging process affects Parkinson’s disease patients more than the general population.

The absence of a standardized way to evaluate lower-limb performance in the PD population preclude any comparison with other studies while the clinically oriented measured spinopelvic parameters of this cohort of patients are similar to what has been published in previous studies [20,22,26]. Data about spinal alignment can be considered representative of the average PD population observed, and we suggest that the assessment protocol presented in this study could be introduced in a cost-effective manner in a clinical setting.

The absolute (Watt) and relative power production (Watt/Kg), as measured by the Muscle Quality Index test, do not correlate with the amount of METs produced weekly in both groups. In our opinion, this stresses the concept that non-specific physical activity does not make a relevant difference in preventing muscular quality deterioration in both PD patients and controls. Specific exercise programs should rely upon high-intensity interval training concepts in aiming to obtain the best possible advantages from the amount of time spent in physical activity during the week [27,28]. The exercises of an HIIT program could specifically select to train hip and knee extensors and, more generally, the muscle groups involved in a sit-to-stand task, static balance and dynamic balance. Clinicians could eventually use the individual peak power value measured during the MQI test to set the submaximal overload according HIIT protocol [27,28]. In this sense, we consider the MQI test as a clinically oriented tool to reach the best specificity of HIIT for any single patient, as clinician can use this low-cost performance test also in an outpatient setting in a very fast way during the standard medical examination. MQI provides also a standardized and scientifically acceptable way to ensure a precise during the follow up of neurodegenerative and chronic diseases.

The adoption of such a precise assessment and training protocol will also, at least partially, overcome the negative atrophic process of lower limbs’ muscle masses (reduction in muscle quantity) and the increase in intramuscular fat and other non-contractile tissue (reduction in muscle quality) as observed in the aging process. Although qualitative changes in the lower extremities were reported to be a factor linked to muscle strength reduction in different cohorts of subjects [27,28,29,30], to the best of our knowledge, the quantitative changes in lower-limb power related to spinal posture have been little explored so far in PD patients.

Therefore, the ability to develop strength and power (the amount of energy transferred or converted per unit time) should be addressed by a specifically designed strength and power training program designed to reduce the impact of degenerative changes in muscular tissue in the trunk and lower extremities’ muscles [30].

Clinicians should pay great attention to these concepts because, as observed in the results of the present study, at least 14% of the examined PD population is at risk of independence loss in daily life activities, while 44% of them could rapidly overcome the critical threshold of 0.9 Watt/kg.

Furthermore, in a recently published study, the absolute peak power measured by MQI in repeated sit-to-stand tasks has the ability to discriminate between the subgroups of patients with the highest risk of mortality in the long term [23]. According to these results, clinicians should pay more attention and dedicate more time to patients who are not able to overcome the cut-off value of 118 Watts, as proposed by Brown et al. [23].

According to our results, in the clinical evaluation, it is mandatory to consider sagittal balance at least in the measure of C7 plumb line as a factor potentially related to lower limbs’ muscle power reduction. L3, S1 and kyphosis apex plumb lines, should be also measured to obtain a global perspective of the spinal alignment. Not only linear parameters but also angular values should be considered, because in PD patients, the upper thoracic inclination of kyphosis (T1-T2 angle) and sagittal plumb lines of C7 and kyphosis apex were significantly higher than in the control group (*p* < 0.05).

### Limits of the Study

Our study presents some limitations. The PD patients and the CON group were not perfectly matched by age (6–7 years of difference), and the mean age of the PD group is 75 years, which is a bias when evaluating PD itself. Another limitation is the measurement of weekly physical activity using IPAQ classification, which is a not a precise tool to quantify physical and recreational activity. However, to minimize overestimation errors due to self-administration of the questionnaire, in our study, the same operator collected all the responses.

## 5. Conclusions

Our results lead to the possibility that the preservation of lumbar lordosis may be one of the factors necessary to maintain efficient biomechanics of the lower-limb muscles. More than non-specific physical activity, the conservation of physiological contractile characteristics of these muscles can probably be mostly susceptible to a specific multidisciplinary rehabilitation intervention based on postural exercises of the spine and specifically designed exercise programs to stimulate power production in lower-limb muscles.

## Figures and Tables

**Table 1:** Descriptive statistics and functional scores of healthy controls and Parkinson’s disease patients.

	Healthy Controls (*n* = 42)			Parkinson Patients (*n* = 43)			
	Median	Q1	Q3	Median	Q1	Q3	*p*-Value
Age	68.9	65.4	73.3	75.3	67.2	78.8	0.01 *
Height (cm)	166.8	160.0	171.0	165.0	157.0	172.0	0.36
Weight (kg)	76.0	63.0	84.0	72.0	63.0	84.0	0.44
BMI	26.9	24.0	29.1	27.2	22.9	29.4	0.80
METs/wk	1140.0	570.0	2625.0	910.0	187.5	1670.0	0.07
Sit to stand time (s)	20.5	17.0	25.0	24.5	21.0	35.5	<0.01
MQI (Watt)	135.9	110.5	177.7	101.5	69.1	143.0	<0.01 *
MQI (Watt/kg)	1.9	1.5	2.4	1.5	1.0	1.7	<0.01 *

BMI: Body Mass Index; ATR: Angle of Trunk Rotation; METs/wk: weekly Metabolic Equivalents. * Statistically significant at α = 0.05.

**Table 2:** Parameters of spinal alignment in healthy controls and Parkinson’s disease patients.

	Healthy Controls (*n* = 42)			Parkinson Patients (*n* = 43)			*p*-Value
	Median	Q1	Q3	Median	Q1	Q3	
ATR (°)	3.5	2.0	5.0	4.0	0.0	5.0	0.74
PL-C7 (mm)	55.0	50.0	80.0	100.0	80.0	130.0	<0.01 *
PL-AK (mm)	0.0	0.0	0.0	0.0	0.0	25.0	<0.01 *
PL-L3 (mm)	40.0	30.0	50.0	40.0	30.0	50.0	0.26
PL-S1 (mm)	10.0	0.0	20.0	0.0	0.0	20.0	0.02 *
Inclinometer C7-T1 (°)	64.0	57.0	70.0	54.0	45.0	59.0	<0.01 *
Inclinometer D12-L1 (°)	81.0	76.0	86.0	84.0	80.0	87.0	0.07

ATR: Angle of Trunk Rotation; PL-C7: Plumb line on spinous process of C7; PL-AK Plumb line on apex of kyphosis; PL-L3: Plumb line on spinous process of L3; PL-S1: Plumb line on sacrum. * Statistically significant at α = 0.05.

**Table 3:** Correlations of Muscle Quality Index and plumb lines in healthy controls and Parkinson’s disease patients.

	Healthy Controls (*n* = 42)		Parkinson Patients (*n* = 43)	
	ρ	*p*-Value	ρ	*p*-Value
MQI vs. PL-C7	−0.01	0.94	0.02	0.88
MQI vs. PL-AK	−0.08	0.64	−0.02	0.91
MQI vs. PL-L3	0.24	0.12	0.35	0.03 *
MQI vs. PL-S1	0.11	0.50	0.04	0.82

MQI: Muscle Quality Index; PL-C7: Plumb line on spinous process of C7; PL-AK Plumb line on apex of kyphosis; PL-L3: Plumb line on spinous process of L3; PL-S: Plumb line on sacrum. * Statistically significant at α = 0.05.

## Data Availability

The data presented in this study are available on request from the corresponding authors.

## References

[1] Ashour R., Jankovic J. (2006). Joint and skeletal deformities in Parkinson’s disease, multiple system atrophy, and progressive supranuclear palsy. Mov. Disord. Off. J. Mov. Disord. Soc..

[2] Srivanitchapoom P., Hallett M. (2016). Camptocormia in Parkinson’s disease: Definition, epidemiology, pathogenesis and treatment modalities. J. Neurol. Neurosurg. Psychiatry.

[3] Baik J.S., Kim J.Y., Park J.H., Han S.W., Park J.H., Lee M.S. (2009). Scoliosis in patients with Parkinson’s disease. J. Clin. Neurol..

[4] Doherty K.M., van de Warrenburg B.P., Peralta M.C., Silveira-Moriyama L., Azulay J.P., Gershanik O.S., Bloem B.R. (2011). Postural deformities in Parkinson’s disease. Lancet. Neurol..

[5] Ameghino L., Rossi M., Merello M. (2016). How Do I Examine Postural Disorders in Parkinson’s Disease?. Mov. Disord. Clin. Pract..

[6] Hasegawa T., Treis A., Patenge N., Fiesel F.C., Springer W., Kahle P.J. (2008). Parkin protects against tyrosinase-mediated dopamine neurotoxicity by suppressing stress-activated protein kinase pathways. J. Neurochem..

[7] Duncan R.P., Leddy A.L., Earhart G.M. (2011). Five times sit-to-stand test performance in Parkinson’s disease. Arch. Phys. Med. Rehabil..

[8] Tanaka K., Quadros A.C., Santos R.F., Stella F., Gobbi L.T., Gobbi S. (2009). Benefits of physical exercise on executive functions in older people with Parkinson’s disease. Brain Cogn..

[9] Margraf N.G., Rohr A., Granert O., Hampel J., Drews A., Deuschl G. (2015). MRI of lumbar trunk muscles in patients with Parkinson’s disease and camptocormia. J. Neurol..

[10] Bissolotti L., Berjano P., Zuccher P., Zenorini A., Buraschi R., Villafane J.H., Negrini S. (2017). Sagittal balance is correlated with Parkinson’s Disease clinical parameters: An overview of spinopelvic alignment on 175 consecutive cases. Eur. Spine J..

[11] Bissolotti L., Isacco-Grassi F., Orizio C., Gobbo M., Berjano P., Villafane J.H., Negrini S. (2015). Spinopelvic balance and body image perception in Parkinson’s disease: Analysis of correlation. Eur. Spine J..

[12] Ito H., Yokoi D., Kobayashi R., Okada H., Kajita Y., Okuda S. (2020). The relationships between three-axis accelerometer measures of physical activity and motor symptoms in patients with Parkinson’s disease: A single-center pilot study. BMC Neurol..

[13] Monjo H., Fukumoto Y., Asai T., Shuntoh H. (2018). Muscle Thickness and Echo Intensity of the Abdominal and Lower Extremity Muscles in Stroke Survivors. J. Clin. Neurol..

[14] Takai Y., Ohta M., Akagi R., Kanehisa H., Kawakami Y., Fukunaga T. (2009). Sit-to-stand test to evaluate knee extensor muscle size and strength in the elderly: A novel approach. J. Physiol. Anthropol..

[15] Akbar U., McQueen R.B., Bemski J., Carter J., Goy E.R., Kutner J., Johnson M.J., Miyasaki J.M., Kluger B. (2021). Prognostic predictors relevant to end-of-life palliative care in Parkinson’s disease and related disorders: A systematic review. J. Neurol. Neurosurg. Psychiatry.

[16] Rainoldi L., Zaina F., Villafañe J.H., Donzelli S., Negrini S. (2015). Quality of Life in Normal and Idiopathic Scoliosis Adolescents before diagnosis: Reference values and discriminative validity of the SRS-22. A cross-sectional study of 1205 pupils. Spine J..

[17] Hasegawa R., Islam M.M., Lee S.C., Koizumi D., Rogers M.E., Takeshima N. (2008). Threshold of lower body muscular strength necessary to perform ADL independently in community-dwelling older adults. Clin. Rehabil..

[18] Inkster L.M., Eng J.J., MacIntyre D.L., Stoessl A.J. (2003). Leg muscle strength is reduced in Parkinson’s disease and relates to the ability to rise from a chair. Mov. Disord. Off. J. Mov. Disord. Soc..

[19] Mak M.K., Hui-Chan C.W. (2002). Switching of movement direction is central to parkinsonian bradykinesia in sit-to-stand. Mov. Disord. Off. J. Mov. Disord. Soc..

[20] Nocera J.R., Buckley T., Waddell D., Okun M.S., Hass C.J. (2010). Knee extensor strength, dynamic stability, and functional ambulation: Are they related in Parkinson’s disease?. Arch. Phys. Med. Rehabil..

[21] Bissolotti L., Gobbo M., Villafane J.H., Negrini S. (2014). Spinopelvic balance: New biomechanical insights with clinical implications for Parkinson’s disease. Eur. Spine J..

[22] Nakamura Y., Machida Y., Hanawa H., Kanai M., Asano S. (2019). Analysis of Relationships between Spinal Deformity and Walking Ability in Parkinson’s Disease Patients. Spine Surg. Relat. Res..

[23] Brown J.C., Harhay M.O., Harhay M.N. (2016). The muscle quality index and mortality among males and females. Ann. Epidemiol..

[24] Nascimento D.D.C., Prestes J., de Sousa Diniz J., Beal P.R., Alves V.P., Stone W., Beal F.L.R. (2020). Comparison of field- and laboratory-based estimates of muscle quality index between octogenarians and young older adults: An observational study. J. Exerc. Rehabil..

[25] Lee C., Dierickx E. (2018). Defining Sarcopenia Using Muscle Quality Index. J. Aging Res. Clin. Pract..

[26] Bissolotti L., Donzelli S., Gobbo M., Zaina F., Villafane J.H., Negrini S. (2016). Association Between Sagittal Balance and Scoliosis in Patients with Parkinson Disease: A Cross-sectional Study. Am. J. Phys. Med. Rehabil..

[27] Schenkman M., Moore C.G., Kohrt W.M., Hall D.A., Delitto A., Comella C.L., Josbeno D.A., Christiansen C.L., Berman B.D., Kluger B.M. (2018). Effect of High-Intensity Treadmill Exercise on Motor Symptoms in Patients With De Novo Parkinson Disease: A Phase 2 Randomized Clinical Trial. JAMA Neurol..

[28] Moreno Catala M., Woitalla D., Arampatzis A. (2013). Central factors explain muscle weakness in young fallers with Parkinson’s disease. Neurorehabilit. Neural Repair.

[29] Alberts J.L., Rosenfeldt A.B., Lopez-Lennon C., Suttman E., Jansen A.E., Imrey P.B., Dibble L.E. (2021). Effectiveness of a Long-Term, Home-Based Aerobic Exercise Intervention on Slowing the Progression of Parkinson Disease: Design of the Cyclical Lower Extremity Exercise for Parkinson Disease II (CYCLE-II) Study. Phys Ther..

[30] Lima L.O., Cardoso F., Teixeira-Salmela L.F., Rodrigues-de-Paula F. (2016). Work and power reduced in L-dopa naive patients in the early-stages of Parkinson’s disease. Arq. Neuro-Psiquiatr..

